# A role for the spindle assembly checkpoint in the DNA damage response

**DOI:** 10.1007/s00294-016-0634-y

**Published:** 2016-08-03

**Authors:** Roger Palou, Gloria Palou, David G. Quintana

**Affiliations:** Biophysics Unit, School of Medicine, and Department of Biochemistry and Molecular Biology, Universitat Autonoma de Barcelona, Bellaterra, 08193 Catalonia, Spain

**Keywords:** DNA damage response (DDR), Spindle assembly checkpoint (SAC), S Phase checkpoint, Chromosome segregation, Genomic instability, Cyclin Dependent Kinase (Cdk1)

## Abstract

Spontaneous DNA damage poses a continuous threat to genomic integrity. If unchecked, genotoxic insults result in genomic instability, a hallmark of cancer cells. In eukaryotic cells a DNA Damage Response (DDR) detects and responds to genotoxic stress, acting as an anti-cancer barrier in humans. Among other actions, the DDR blocks the segregation of incompletely replicated or damaged chromosomes, thus preventing aneuploidy. In a work aimed at better understanding such S-M control, we recently showed that cells block anaphase through different control pathways. The S phase checkpoint kinase Mec1/ATR inhibits mitotic Cyclin Dependent Kinase activity through effector kinases Swe1/Wee1 and Rad53/Chk2. Cells also stabilize the levels of Pds1/securin to block sister chromatid segregation in response to DNA damage. We show here that Pds1/securin abundance is still secured when the S phase checkpoint response is fully abrogated in *mec1/ATR tel1/ATM* double null mutants. When such cells are exposed to genotoxic stress, Pds1/securin is stabilized in a spindle assembly checkpoint (SAC) dependent manner. Disruption of the SAC and the S phase checkpoint together, allows chromosome segregation in the presence of DNA damage or replication stress. Our results place the SAC as a part of the DDR, which appears to count on different, independent control layers to preserve genomic integrity when chromosome replication is challenged.

## Introduction

Cells are continuously exposed to spontaneous DNA damage, mostly due to hydrolysis and oxidation of bases, a natural payoff of life based on water and oxygen. If not taken care of, the presence of lesions in the DNA leads to genomic instability, due to replication errors, accumulation of mutations, and chromosome sections left unreplicated due to collapsed replication forks. To prevent such outcome, eukaryotic cells count on the so-called DNA damage response (DDR), that detects and responds to insults that challenge chromosome replication. Among other actions, the DDR blocks the segregation of incompletely replicated or damaged chromosomes, thus preventing aneuploidy. As expectable, in humans the DDR acts as an anti-cancer barrier in early tumorigenesis (Barktova et al. 2005, 2006; Gorgoulis et al. 2005; Bartek et al. 2007).

A central component of the DDR is the so-called S phase checkpoint. The S phase checkpoint responds to insults that threaten chromosome replication, such as DNA damage or the shortage of deoxynucleotides. In response to such challenges, the S phase checkpoint blocks mitotic chromosome segregation (Weinert and Hartwell 1988) and slows down DNA replication (Paulovich and Hartwell 1995).

Checkpoints are highly conserved surveillance mechanisms that play a critical role to preserve genomic integrity in eukaryotic cells. Checkpoints characteristically ensure that critical cell cycle events are successfully completed before progression to a subsequent phase is allowed. Loss of checkpoint function results in genomic instability (Hartwell et al. 1994), which is the driving force that fuels cancer transformation (Cahill et al. 1999; Gatenby and Gillies 2008). Mechanistically, checkpoints are signal transduction pathways triggered by intracellular signals, and are constituted by sensor complexes, central transducer kinases, and downstream effector kinases (Zhou and Elledge 2000). Mec1, the ortholog of human ATR, is the S phase checkpoint central transducer kinase in the budding yeast *Saccharomyces cerevisiae*. Paralog kinase Tel1/ATM may partially replace Mec1/ATR under some conditions. Mec1/ATR activates effector kinases Chk1 and Rad53, the yeast ortholog of human Chk2.

Another checkpoint, the Spindle Assembly Checkpoint (SAC), blocks progression to anaphase until each and every chromosome is attached to the spindle and under bipolar tension (Rieder et al. 1995; Vanoosthuyse and Hardwick 2009), thus preventing the occurrence of unbalanced chromosome segregation and aneuploidy. The SAC blocks anaphase by keeping inactive the ubiquitin ligase APC^Cdc20^, essential to target Pds1/securin for degradation (Hardwick and Murray 1995). Pds1/securin is a chaperone that inhibits the protease Esp1/separase. In turn, Esp1/separase is responsible for the cleavage of cohesin required for sister chromatid segregation (Yamamoto et al. 1996; Ciosk et al. 1998: Uhlmann et al. 1999). APC^Cdc20^ is also required for the eventual release of the Cdc14 phosphatase, essential for mitotic exit (Machin et al. 2016).

In our recent work to better understand how cells block anaphase in response to challenged DNA replication, we showed that the S phase checkpoint prevents chromosome segregation through three independent, redundant, downstream pathways (Palou et al. 2015). Mec1/ATR inhibits mitotic Cdk1 activity through downstream effector kinases, Rad53/Chk2 and Swe1/Wee1. However, deletion of Rad53/Chk2 and Swe1/Wee1 is not enough to allow cells slip into anaphase in the presence of genotoxic stress, and control on Pds1/cohesin must be abrogated as well (Palou et al. 2015). Previous reports place Pds1/securin under the control of the checkpoint effector kinases Chk1 and Rad53/Chk2 (Sanchez et al. 1999; Agarwal et al. 2003; Kim and Burke 2008).

## Chromosome segregation in response to genotoxic stress is still blocked when Mec1/ATR and Tel1/ATM signaling is abrogated

As described above, based on available knowledge, the whole S-M control relies on the S phase checkpoint. One prediction arising from this model is that cells deleted for Mec1/ATR, the DNA damage response central transducer kinase, should fail to block anaphase in the presence of genotoxic stress. We started testing such prediction by exposing *mec1* null cells to DNA damage during S phase.

**Fig. 1:**
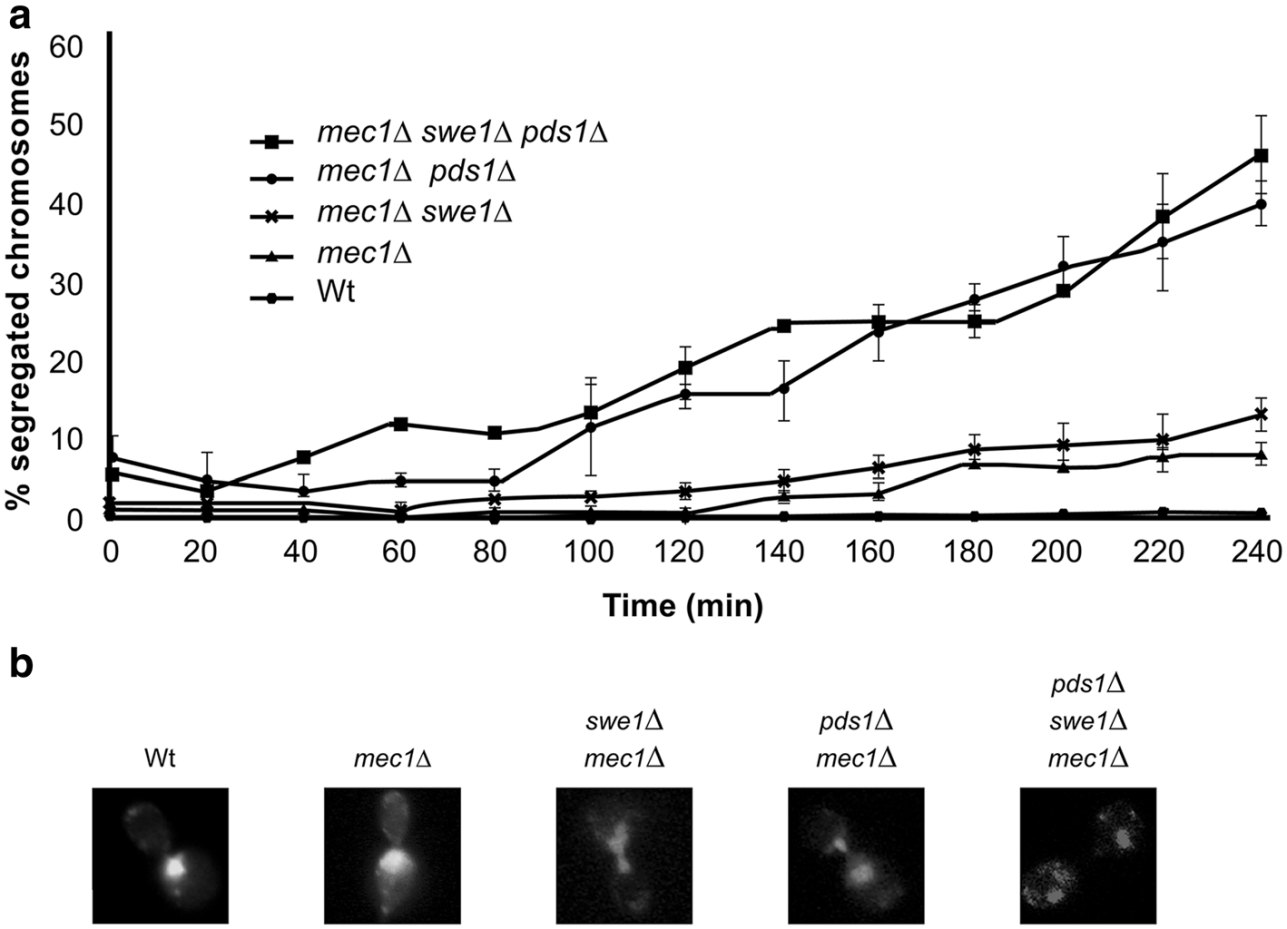
Deletion of Mec1 is not sufficient to allow the segregation of damaged chromosomes. Cells with the indicated genotypes were grown to mid-exponential phase, synchronized in G1 phase with the pheromone alpha-factor, and released into S phase in the presence of 0.033 % MMS. Cells were collected at the indicated times (min). All strains are *sml1*∆ isogenic, to rescue the lethality of the Mec1 deletion. **a** Percentage of cells showing chromosome segregation. 120 cells were counted in 3 independent experiments. The results are expressed as mean ± standard deviation. **b** DAPI stained nuclei were visualized by means of fluorescence microscopy. Representative cells are shown for the indicated genotypes 4 h after release from G1

Exponentially growing cells were synchronized in pre-Start G1 phase. Cells were then synchronously released into S phase in the presence of the DNA methylating reagent methyl methanesulfonate (MMS). Samples were collected at different times during 4 h. Collected cells were fixed, stained with DAPI, and chromosomes were visualized by means of fluorescence microscopy. Strikingly, cells lacking Mec1/ATR remain competent to block chromosome segregation in the presence of DNA damage (Fig. 1). Deletion of the Mec1/ATR effector Swe1/Wee1 (Palou et al. 2015) made no difference.

**Fig. 2:**
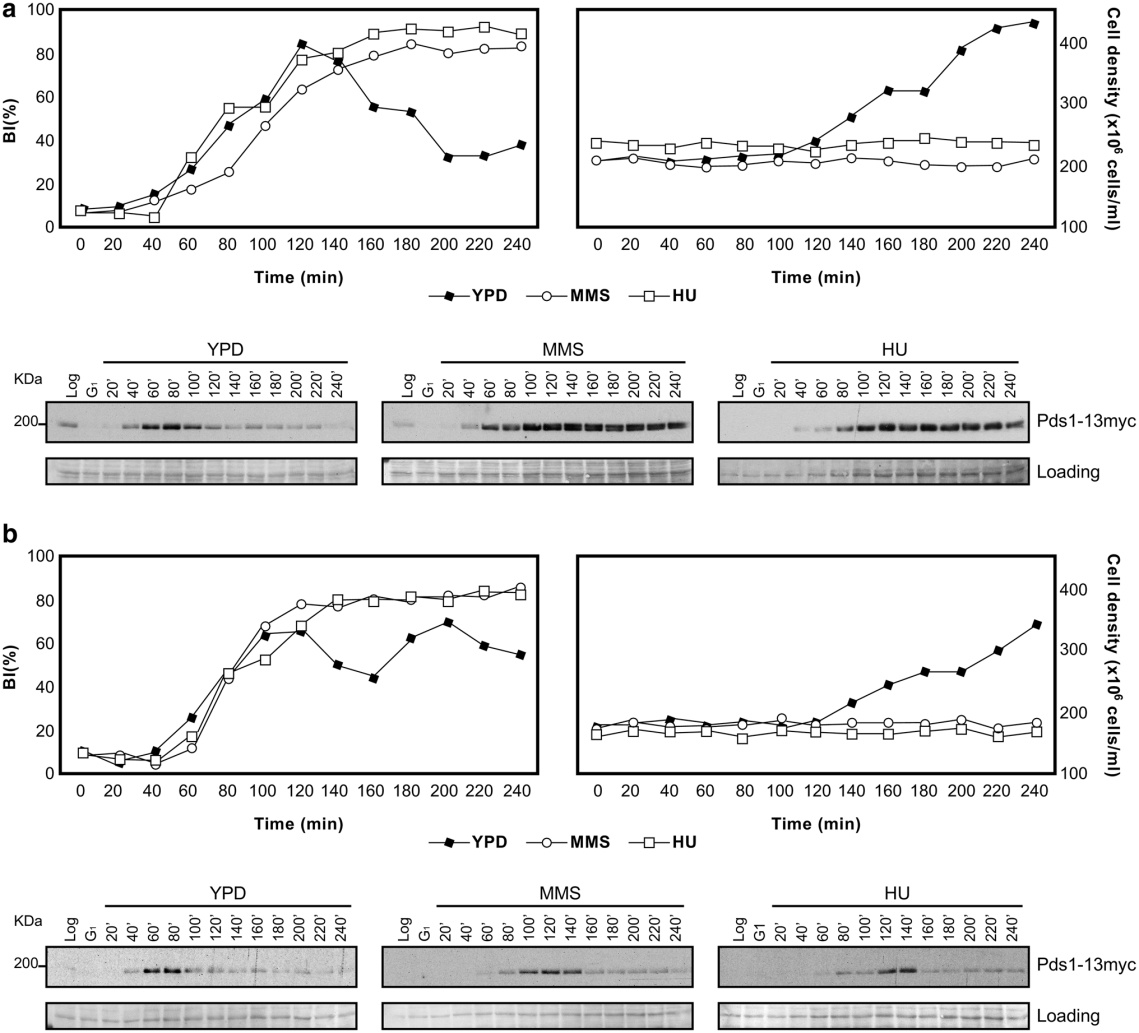
Levels of Pds1/securin in the presence of genotoxic stress in S phase checkpoint and SAC mutants. Cultures of *mec1*∆ *tel1*∆ cells (**a**) or *mec1*∆ *tel1*∆ *mad2*∆ cells (**b**) were grown to mid-exponential phase, synchronized in G1 phase with the pheromone alpha-factor, then released into S phase in the presence of 0.2 M HU, 0.033 % MMS, or in the absence of genotoxic stress (YPD). Cells were collected at the indicated times (min). All strains are *sml1*∆ isogenic, to rescue the lethality of the Mec1 deletion. As a measure of synchronicity and cell cycle progression, the *upper panels* show the budding indexes (BI  %) and cell densities of the cultures (average of 3 independent experiments). The* lower panels* show representative Pds1/securin immunoblots on whole cell extracts. A Ponceau S-stained region of the same membrane used for Western blotting is shown as a loading control

Since deletion of Mec1/ATR is sufficient to abrogate the regulation of mitotic Cdk1 activity (Palou et al. 2015), we next checked the levels of Pds1/securin, the third S-M control branch. To discard a contribution from the Mec1/ATR paralog kinase Tel1/ATM, both kinases were deleted this time. As shown in Fig. 2a, the double deletion mutant *tel1*∆ *mec1*∆ is still able to keep stable levels of Pds1/securin when exposed to DNA damage during S phase (MMS) or to replication stress (hydroxyurea, HU). These results indicate that an alternative control, independent of Mec1/ATR and Tel1/ATM signaling, avoids that Pds1/securin is removed when cells are exposed to genotoxic stress.

## The SAC blocks chromosome segregation in response to genotoxic stress in a Mec1/ATR and Tel1/ATM independent manner

We, therefore, wished to explore whether the Spindle Assembly Checkpoint (SAC) is responsible for the stable levels of Pds1/securin in absence of Mec1/ATR and Tel1/ATM signaling. The SAC central element Mad2 was deleted in a *mec1*∆ *tel1*∆ background and the triple mutant was exposed to genotoxic stress. Indeed, *mec1*∆ *tel1*∆ *mad2*∆ cells exposed to replication stress (HU) or to DNA methylation damage (MMS) fail to keep stable levels of Pds1/securin (Fig. 2b). Therefore, the SAC on its own stabilizes Pds1/securin levels in cells exposed to genotoxic stress in S phase.

**Fig. 3:**
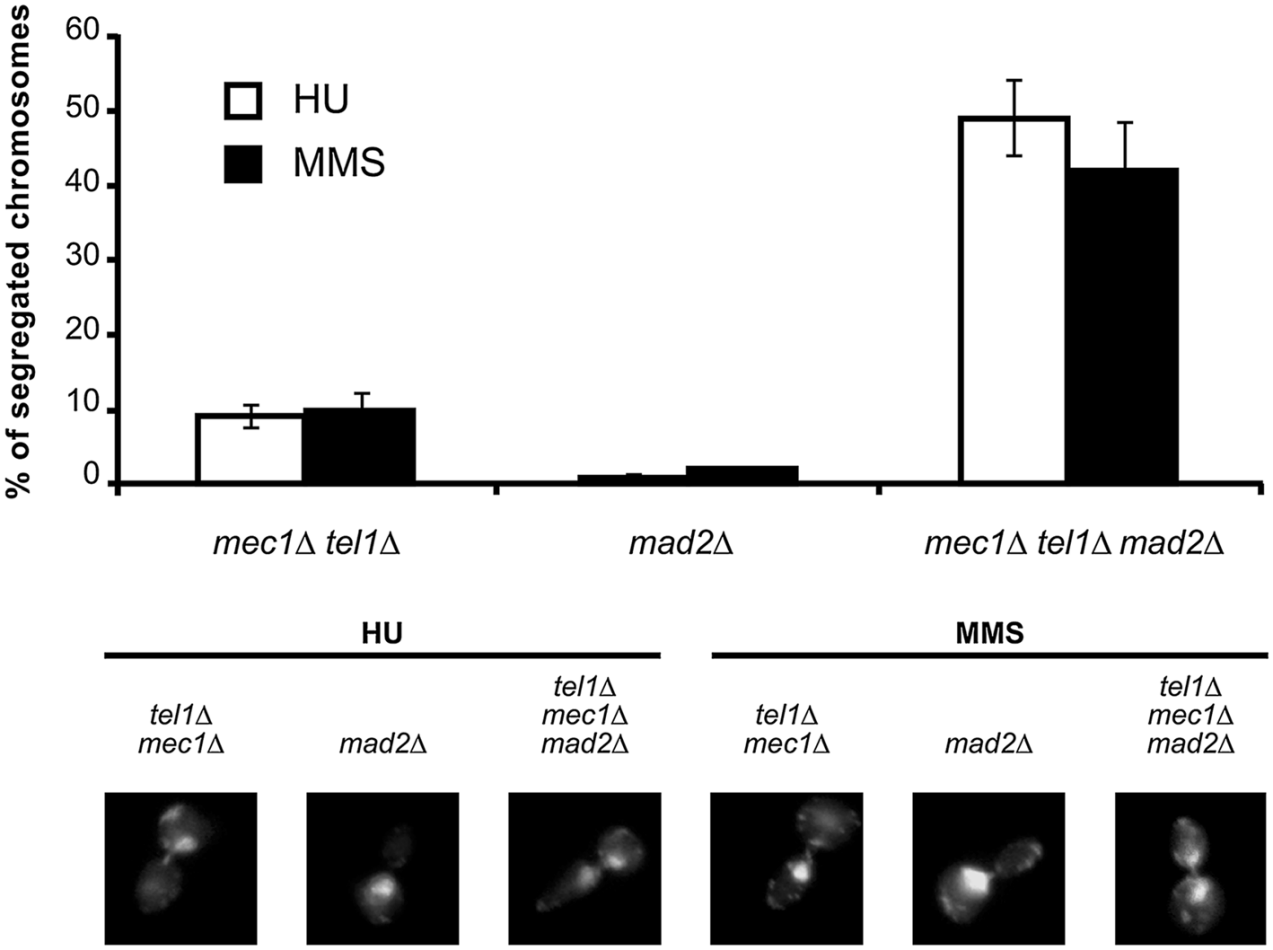
The S phase checkpoint and the SAC redundantly block chromosome segregation in the presence of DNA damage. Cells with the indicated genotypes were grown to mid-exponential phase, synchronized in G1 phase with the pheromone alpha-factor, then released into S phase in the presence of 0.2 M HU or 0.033 % MMS. Cells were collected 4 h after release from G1. All strains are *sml1*∆ isogenic, to rescue the lethality of the Mec1 deletion. *Upper panel* Percentage of cells showing chromosome segregation. 120 cells were counted in two independent experiments. The results are expressed as mean ± standard deviation.* Lower panel* DAPI stained nuclei were visualized by means of fluorescence microscopy. Representative cells are shown for the indicated genotypes 4 h after release from G1

In agreement with the loss of control on Pds1/securin, loss of the SAC and the S phase checkpoint allows *mec1*∆ *tel1*∆ *mad2*∆ *PDS1* ^+^ cells to enter anaphase in the presence of genotoxic stress (Fig. 3). On the contrary, abrogation of the S phase checkpoint alone, or the SAC alone, is not sufficient.

## SAC as part of the DDR

In summary, our results place the Spindle Assembly Checkpoint as part of the DNA Damage Response. In the absence of Mec1/ATR and Tel1/ATM signaling the SAC is still able, and becomes essential, to block the segregation of incompletely replicated chromosomes.

Previous reports had placed the stabilization of Pds1/securin levels in response to DNA damage under the Mec1/ATR downstream effector kinases Chk1 and Rad53/Chk2 (Sanchez et al. 1999; Agarwal et al. 2003; Kim and Burke 2008). However, we show here that cells are still able to keep Pds1/securin levels stable in response to genotoxic stress in the absence of Mec1/ATR and Tel1/ATM. The observations in previous reports and in our work may be reconciled based on the distinct genotoxic scenarios in the different studies. Mec1/ATR and Tel1/ATM signaling may indeed be essential for the stabilization of Pds1/securin levels when DNA damage is sensed in G2 phase (Sanchez et al. 1999; Agarwal et al. 2003), or in the presence of very low levels of DNA damage that allow the completion of chromosome replication (Kim and Burke 2008). In those cases, chromosomes should be able to attach to the spindle and undergo the bipolar tension that inactivates the SAC. On the contrary, cells exposed to replication stress or to significant levels of DNA damage during S phase cannot complete chromosome replication. In such scenario, chromosomes that fail to replicate centromeric DNA will be unable to engage in bipolar attachment to the spindle. In that case, the SAC will remain active, APC^Cdc20^ inactive, Pds1/securin abundance stable, and anaphase blocked. As a result, S phase checkpoint signaling becomes dispensable to block chromosome segregation. In such explanatory model, the SAC acts merely as a serendipitous backup, and does not require cross-talk with the S-phase checkpoint. However, in future work it will be of interest to explore whether the two checkpoints are indeed mutually wired.

Our observation is in fair agreement with a previous observation showing that Pds1/securin stabilization upon recovery from replication stress is largely alleviated in a *rad53 mad2*∆ double mutant, but not when only *rad53* is mutated (Feng et al. 2009). The authors suggest that SAC activation may result from defective bi-orientation of sister chromatids, in turn due to unreplicated centromeres in *rad53* mutant cells recovering from transient exposure to replication stress. That may as well be the case in our checkpoint mutant strains.

**Fig. 4:**
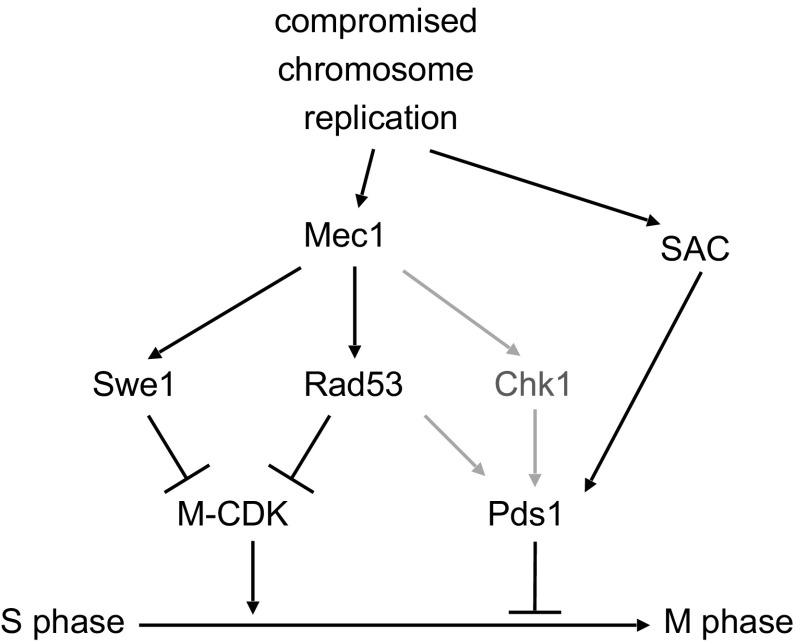
The Spindle Assembly Checkpoint contributes to the DNA Damage Response. Molecular diagram showing the pathways that block anaphase in response to genotoxic stress. Our results place the SAC as a redundant control that blocks chromosome segregation even in the absence of an S phase checkpoint response. In grey, regulatory pathway taken from previous works

An integrated model, resulting from our previous work (Palou et al. 2015) and this report is summarized in Fig. 4. Our results place the SAC as an additional layer of control in the DDR that prevents the segregation of incompletely replicated or damaged chromosomes. Multiple pathways appear to redundantly contribute to the critical S-M control that prevents aneuploidy when S phase is challenged by genotoxic stress. Notably, the SAC alone is able to prevent chromosome segregation in the presence of DNA damage in cells lacking Mec1/ATR and Tel1/ATM function. Derived from such observation, the SAC emerges as an attractive target for anti-tumoral therapy. As many cancer cells are characteristically defective in ATM/ATR signaling, blocking SAC signaling might help as co-adjuvant treatment in therapies based on DNA damaging drugs, selectively pushing malignant cells into aberrant, inviable anaphases.
